# Rate of Atherosclerosis Progression in ApoE^−/−^ Mice Long After Discontinuation of Cola Beverage Drinking

**DOI:** 10.1371/journal.pone.0089838

**Published:** 2014-03-26

**Authors:** Matilde Otero-Losada, Gabriel Cao, Santiago Mc Loughlin, Gastón Rodríguez-Granillo, Graciela Ottaviano, José Milei

**Affiliations:** Instituto de Investigaciones Cardiológicas, Universidad de Buenos Aires, Consejo Nacional de Investigaciones Científicas y Técnicas, ININCA.UBA.CONICET, Buenos Aires, Argentina; University of Buenos Aires, Faculty of Medicine. Cardiovascular Pathophysiology Institute., Argentina

## Abstract

This study was conducted in order to evaluate the effect of cola beverages drinking on atherosclerosisand test the hypothesis whether cola beverages consumption at early life stages might affect the development and progression of atherosclerosis later in life.

ApoE^−/−^ C57BL/6J mice (8 week-old) were randomized in 3 groups (n = 20 each) according to free accessto water (W), sucrose sweetened carbonated cola drink(C) or aspartame-acesulfame K sweetened carbonated ‘light’ cola drink (L)for the next 8 weeks. Drinking treatment was ended by switching C and L groups to drinking water. Four mice per group and time were sequentially euthanized: before treatment (8weeks-old), at the end of treatment (16 weeks-old) and after treatment discontinuation (20 weeks-old, 24 weeks-old, 30 week-old mice). Aortic roots and livers were harvested, processed for histology and serial cross-sections were stained. Aortic plaque area was analyzed and plaque/media-ratio was calculated.

Early consumption of cola drinks accelerated atherosclerotic plaque progression favoring the interaction between macrophages and myofibroblasts, without the participation of either T lymphocytes or proliferative activity. Plaque/media-ratio varied according to drink treatment (F_2,54_ = 3.433, p<0.04) and mice age (F_4,54_ = 5.009, p<0.03) and was higher in C and L groups compared with age-matched W group (p<0.05 at 16 weeks and 20 weeks, p<0.01 at 24 weeks and 30 weeks). Natural evolution of atherosclerosis in ApoE^−/−^ mice (W group) evidenced atherosclerosis acceleration in parallel with a rapid increase in liver inflammation around the 20 weeks of age.

Cola drinking within the 8–16 weeks of age accelerated atherosclerosis progression in ApoE^−/−^ mice favoring aortic plaque enlargement (inward remodeling) over media thinning all over the study time. Data suggest that cola drinking at early life stages may predispose to atherosclerosis progression later in life in ApoE^−/−^ mice.

## Introduction

Atherosclerosis risk increases with age [1] and unhealthy nutritional habits during childhood and youth have been suggested to favor atherosclerosis complications later in life [2], [3]. Longitudinal cohort studies have demonstrated increased cardiovascular risk in adulthood of obese children [4], [5] and that exposure to cardiovascular risk factors early in life may contribute to the development of atherosclerosis [6].The increasing consumption of cola beverages has been associated with obesity and a rising incidence of atherosclerosis and cardiovascular disease over the past decades [7]. We have reported the development of metabolic syndrome after long-term cola beverages consumption in rats [8], [9]. Hypertension, hyperglycemia, increased body weight, dyslipidemia and echocardiographic alterations were observed while pathology findings were scarce, related to aging rather than treatment [8], [9]. Recently we observed that ApoE^−/−^C57BL/6J mice exhibited a particular sensitivity to the effects of cola beverage drinking [10]. Arterial pathology was induced bysucrose-sweetened cola (C) drinking in association with hyperglycemia [10]. Unexpectedly artificially-sweetened ‘light’ cola(L) drinking induced non-metabolic changes compared with water drinking ApoE^−/−^ mice [10]. Both C and L drinkingcaused increase in plaque area(28%C, 50% L) and stenosis (38%C, 57% L) at the end of the drinking period [11]. Discontinuation of cola beverages resulted in paradoxical worsening of lesions (increase in plaque area: 43%C, 68% L andstenosis: 71%C, 46% L). However arterial damage was evaluatedat a single time point after drinking-treatment cessation and the recovery period may have been insufficient to observe reversal of damage. Age as expected was associated to an enlargement of atherosclerotic lesions (56%). Interestingly, treatment- and age-effects on plaque damage were additive [10].

The aim of this study was to further explore the impact of cola beverages drinking on the progression of arterial damagein the ApoE^−/−^ mice model of atherosclerosis [11] t different timeslong after discontinuation of cola beverages drinking-treatment. Concurrently the hypothesis whether consumption of cola beverages early in life might affect the development and progression of atherosclerosis later in life was tested.

## Methods

Ethics Statement: the experiments reported in this study were approved by The Animal Care Committee of the University of Buenos Aires and were performed in accordance with the recommendations of the Weatherall report [12].

SixtyApo E knockout (ApoE^−/−^) mice on a C57BL/6 background (Jackson Laboratory, Bar Harbor, Maine) were fed on a standard rodent commercial chow (Cooperación, Buenos Aires, Argentina) *ad libitum* and housed inside an indoor laboratory facility with a 12 h light/dark cycle.Eight weeks-old ApoE^−/−^ mice were randomized in 3 groups (n = 20 each) according to free access to water (W), regular cola (C) (sucrose sweetened carbonated cola drink, Coca-Cola, Argentina), or light cola (L) (aspartame–acesulfame K sweetened carbonated cola drink, Coca-Cola Light, Argentina) for the next 8 weeks. Drinking treatment was ended by switching C and L groups to drinking water. At the indicated times 4 mice per group and time were sequentially euthanized under anesthesia with sodium pentobarbital and sodium diphenylhydantoin (Euthanyl): before treatment (8weeks-old), at the end of treatment (16 weeks-old) and after treatment discontinuation (20 weeks-old, 24 weeks-old, 30 week-old mice).

Transverse sections from the ascending aorta and liverwere dissected and immersed in 10% buffered formaldehyde (Formalin 10% buffered solution, pH = 7.0) at room temperature for 24 h fixation. After dehydration (ethanol 50%, 70%, 100%) tissues were embedded in paraffin blocks. Serial transverse sections (5 µm) were cut through the aorta at the origins of the aortic valve leaflets throughout the entire aortic sinus and stained with hematoxylin-eosin, Heidenhaintrichrome (Azan) and orcein for elastic fiber identification. Masson's trichrome staining was used to reveal collagen deposition. Immunolabeling of specimens was carried out using a modified avidin–biotin–peroxidase complex technique Vectastain ABC kit (Universal Elite; Vector Laboratories, California, USA). The following antibodies (Santa Cruz Biotechnology Inc., 10410 Finnell Street Dallas, Texas 75220, USA) were used at dilution 1/100: CD3 (PC3/188A) mouse monoclonal antibody to reveal the presence of T-lymphocytes, CD68 (M-20) goat polyclonal antibody to observe macrophages, α-Actin (1A4) mouse monoclonal antibody to identify smooth muscle cellsand PCNA (FL-261, proliferating cell nuclear antigen) rabbit polyclonal antibody to detect proliferative activity.

Each of six serial cross sections was analysed using a software-coupled (Image Pro Plus for Windows, v3) Nikon Eclipse E400 microscope. The macrophage-myofibroblast interaction was estimated by quantitative immunohistochemistry [13]. Plaque area, intimal layer and the media layer length were measured and to account for arterial remodeling, the ratio of the plaque area to the media was calculated (plaque/media-ratio) [14]. Liversections (4 µm) were processed for microscopy and parenchymal inflammation was determined according to non-alcoholic steatohepatitis (NASH) score [15].

Factorial ANOVA was used to identify main sources of data variation, i.e.: drinking treatment and age, followed by the Dunnett post hoc test to assess differences between age-matched experimental groups all over the time of the study. A p<0.05 was considered significant (SPSS version 17.0 software).

## Results

From the qualitative point of view all three groups (W, C, L) were indistinguishable based on morphological characteristics. However, quantitative differences were observed over time depending on drinking treatment.

**Figure 1:**
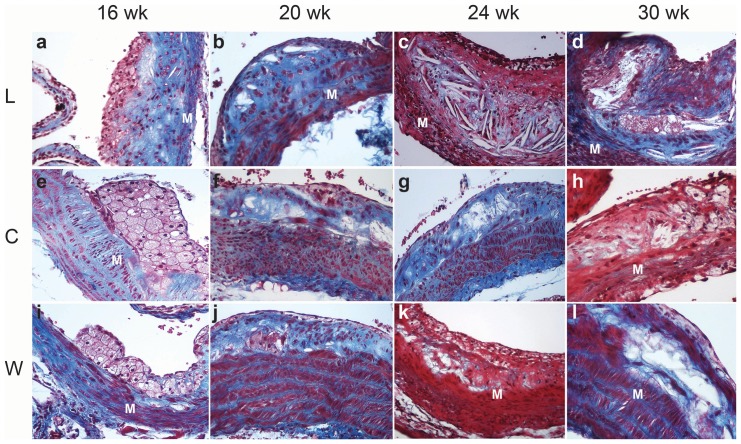
Representative microphotographs of atherosclerotic plaque evolution in L, C and W groups over the study time. Rows (treatment): L, C, W. Columns (age): 16 wk, 20 wk, 24 wk, 30 wk. At the end of treatment: collagen deposits (blue) and cholesterol crystals were observed between clustered foamy macrophages; the thinned subendothelial muscle layer was partially replaced by collagen (M) in the aortic wall in L group (a). Differently C group (e) showed large clusters of subendothelial foamy macrophages over a hypertrophic muscular layer which was partially replaced by collagen (blue). A striking increase in atherosclerotic plaque size relative to muscle layer thickness (M) was observed in L (a–d) compared with C (e–h). Characteristic changes of the natural history of atherosclerotic plaque previously reported in this murine model were found in W group as expected (i, l). Masson's trichrome, original magnification 200×.

**Figure 2:**
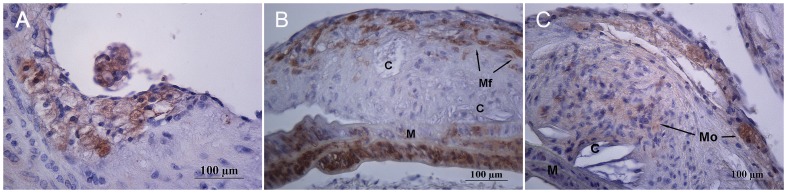
Representative immunohistochemical staining for CD68 and α-smooth muscle actin (α-SMA) illustrating cellular populations involved in plaque formation. A: cytoplasmic immunostaining for CD68 in subendothelial foamy macrophages in 16 week-old mice; B: cholesterol crystals (C), aortic media layer (M) and cytoplasmic expression of α-smooth muscle actin in myofibroblasts (Mf) in 20–24 week-old mice; C: macrophages (Mo) and globular accumulations of lipids in 20–24 week-old mice (C). Original magnification 400×.

At autopsy focal accumulations of subendothelial lipid-laden macrophages expressing CD68 positivity were observed in the vascular intima in 16 weeks-old mice (Figure 1 a, e, I and Figure 2 A, B). Mice 20–24 weeks-old of age showed cytoplasmic expression of smooth muscle α-actin and CD68 indicating the coexistence of myofibroblasts and macrophages respectively in the atherosclerotic plaque (Figure 2B, C). Cholesterol crystals were observed and collagen presence was confirmed by Masson's trichrome staining (Figure 1 b,c–f,g–j,k). Globular accumulations of lipids were covered by a thin fibrous cap. By the 30^th^ week (Figure 1 d, h, l) large acellular necrotic xanthomas were surrounded by collagen material forming a fibro-fatty nodule which extended from the lumen to the internal elastic membrane and largely reducedthe aortic luminal caliber. In mice groups that drank cola beverages,remarkable atrophy of the media layer was observed being replaced with plaque components. Increased cell-proliferative activity (PCNA) or CD3 positivity (T lymphocytes) were not observed in any group.

**Figure 3:**
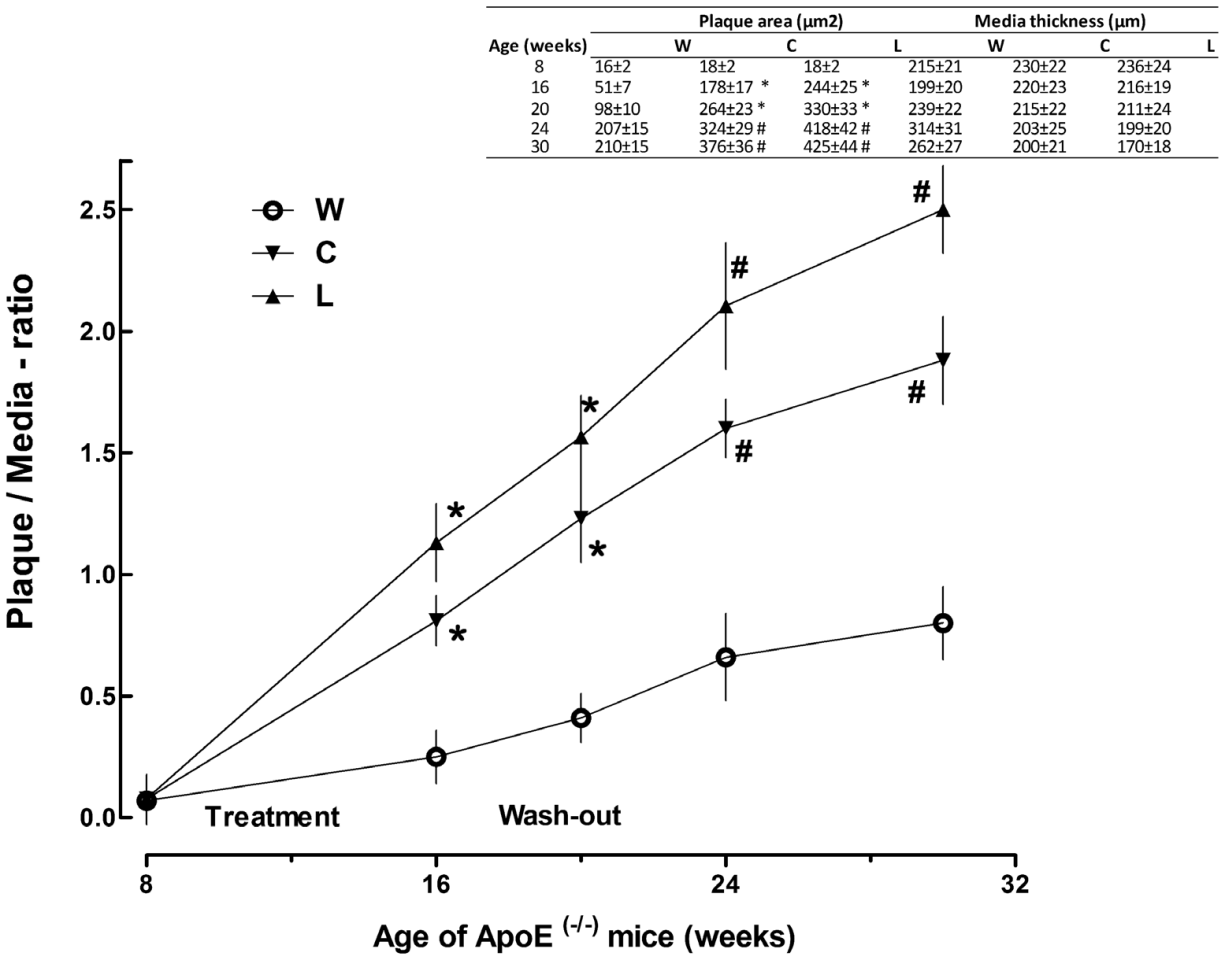
Effect of cola beverages drinking on plaque/media-ratio in ApoE^−/−^ mice over the study time. Inset table shows individual plaque area and media thickness values used to calculate plaque/media-ratio which is plotted in the main graph. *p<0.05, # p<0.01 compared with W.

Regarding quantitative assessment of aortic lesions, plaque/media-ratio varied according to both drink treatment (F_2,54_ = 3.433, p<0.04) and mice age (F_4,54_ = 5.009, p<0.03) as well (Figure 1). Plaque/media-ratio was higher in C and L groups compared with age-matched W group (p<0.05 at 16 weeks and 20 weeks, p<0.01 at 24 weeks and 30 weeks) (Figure 3).

**Figure 4:**
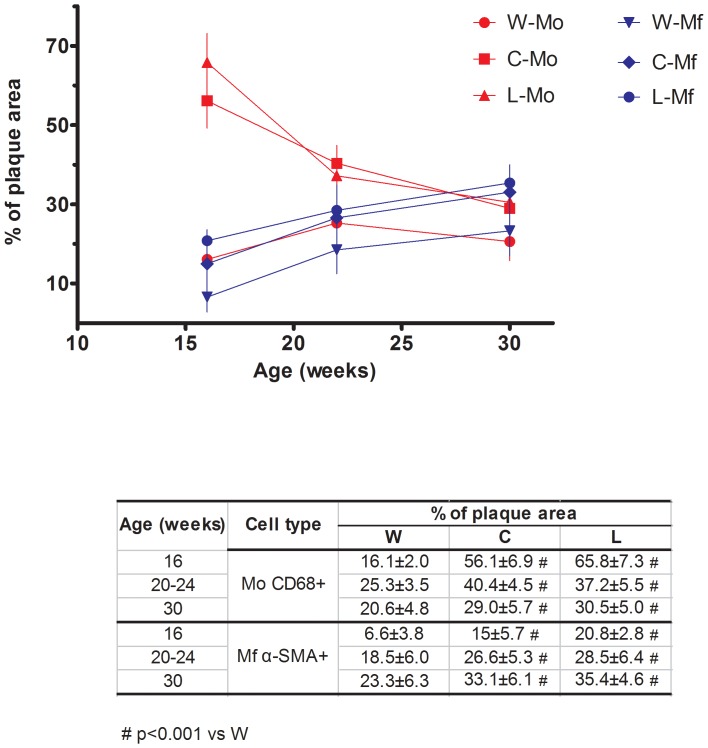
Effect of cola beverages drinking on the population of CD68 positive macrophages (Mo) and α-SMA myofibroblasts (Mf) in atherosclerotic plaques in Apo E^−/−^ mice over the study time. Values are expressed as the mean percentage oftotal plaque area occupied by either CD68 positive macrophages (Mo) or smooth muscle α-actin positive myofibroblasts (Mf). # p<0.001 compared with W.

Figure 4 shows the mean percentage of total plaque area occupied by either CD68 positive macrophages (Mo) or smooth muscle α-actin (α-SMA) positive myofibroblasts (Mf). The mean percentage oftotal plaque area occupied by Mo increased and that of Mf decreased in treated groups compared to control group (p<0.001) at the indicated times during wash-out (see Figure 4, inset table). As a result, Mo and Mf populations matched each other in all groups by the end of the study (30 week-old mice). The natural history of the lesion (control group) showed an increase in Mo population, peaking between the 20^th^ to 24^th^ weeks and declining by week 30 with a converse increase in Mf population.

**Figure 5:**
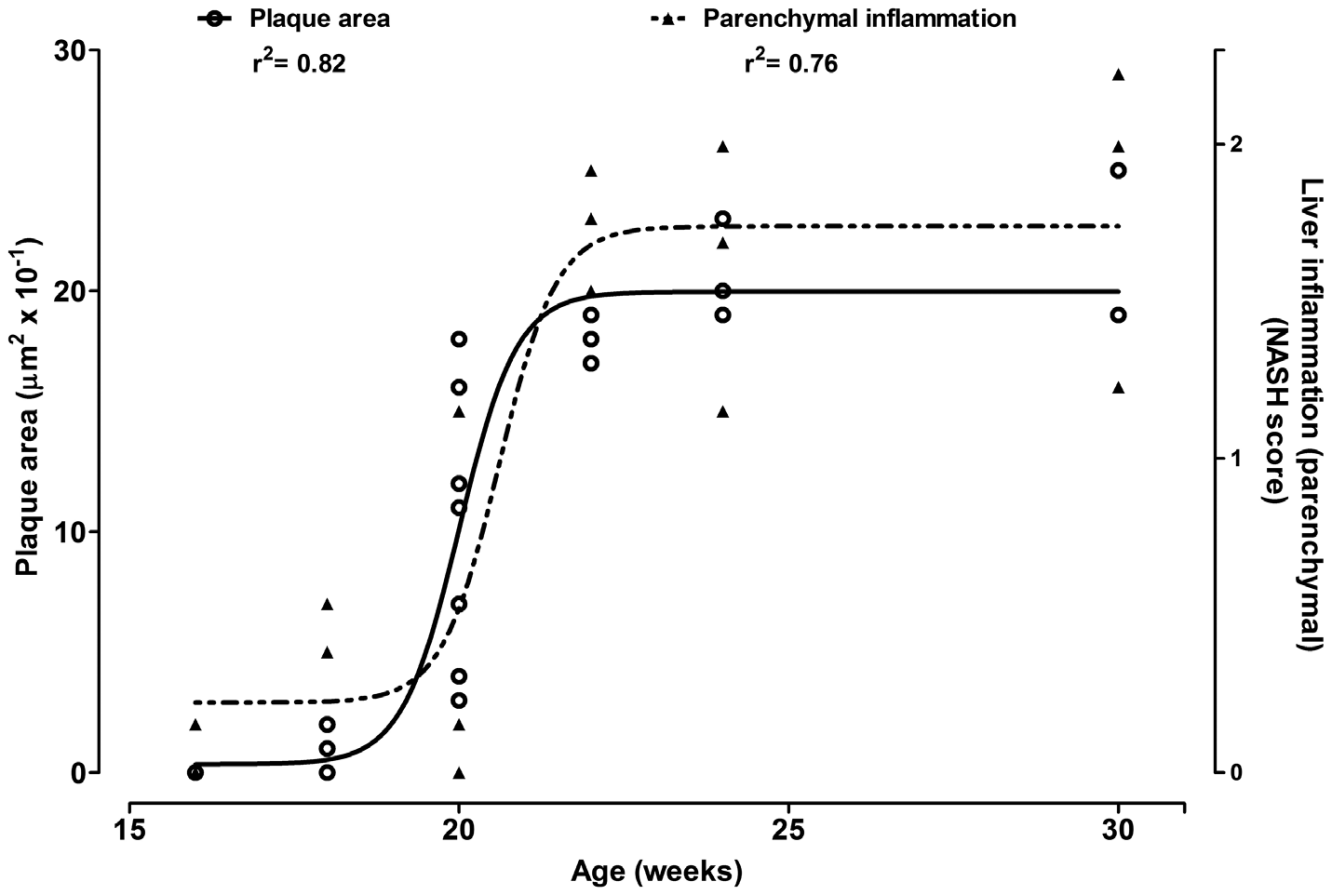
Natural evolution of plaque area and liver inflammation in Apo E^−/−^ mice (water drinking) over time.

ApoE^−/−^ mice spontaneously (i.e.: W group) went through accelerated changes in atherosclerosis progression over time which paralleled a rapid increase in liver inflammation around the 20 weeks of age (Figure 5).

Immunostaining for CD3 and PCNA was performed at weeks 16, 20–24 and 30 using lymph node tissue (Figure S1) and human undifferentiated sarcoma tissue with high mitotic activity (Figure S2) as respective positive controls. Increased cell-proliferative activity (PCNA) or CD3 positivity (T lymphocytes) were not observed in any group.

## Discussion

Rate of progression of atherosclerotic lesions (slopes in Figure 3) was higher in C and L groups compared with W group over the time of the study. Macrophages and myofibroblasts participated in the pathological process while no proliferative activity was found. The observed increase in foamy Mo population in atherosclerotic plaque in ApoE^−/−^ mice might be favored by the characteristic endothelial dysfunction described in this model [11], [16], [17]. During the wash-out period, Mo population decreased likely due to necrosis leading to formation of the large globular accumulations of extracellular lipids observed in 30 week-old mice. At the same time Mo-induced Mf migration into the plaque might contribute with collagen synthesis to fibro fatty nodules formation.Endothelial dysfunction typically observed in ApoE^−/−^ mice [18] is suggested to play a major role in atherosclerotic plaque formation in this mice model long after soft drinks consumption.

Several studies have demonstrated endothelial dysfunction in different vascular beds in ApoE^−/−^ mice [19] and the negative influence of high fat high carbohydrate diets on endothelial function has been confirmed in this mice model. In our analysis, although the pathological sequence leading to atherosclerotic plaque formation was common to all experimental groups, plaque/media-ratio was higher in mice that had consumed cola drinks. These phenomena might be reasonably related to increased expansion and aggravation of endothelial dysfunction opening a new avenue for research.

Previously we reported paradoxical worsening of atherosclerosisat the end of cola beveragesdrinking-treatment [10]. However interpretation of these findings could be questioned considering that determinations had been performed at a single time point immediately after drinking-treatment cessation and the recovery period might have been insufficient to observereversal of damage if any. Present findings confirm that this was not the case indeed. Rate of progression of atherosclerotic lesions increased at different times long after cola drinking-treatment discontinuation. Moreover, the after effects of cola drinking-treatment outweighed those of aging on atherosclerosis time-course. Artificially-sweetened cola drinking (L) accelerated progression of atherosclerosis all over the study time and exceeded the effect of aging when age-matched groups were compared.

Recently we reported an increase in hepatic transaminases activity (2.8 fold), hyperuremia (+74) and hypercreatininemia (2.5 fold) after L drinking in this murine model [10]. Based on these and present findings, we suggest that functional interference at one or more levels (liver, kidney, muscle) and the so derived consequences may be involved in the acceleration of atherosclerosis in ApoE^−/−^ mice observed long after discontinuation of artificially-sweetened cola drinking.

Present findings agree with our recent report [10] and reinforce the idea that ApoE^−/−^ mice may be idiosyncratically sensitive to the effects of cola beverages drinking on arterial damage.

We dare not speculate on possible mechanisms that could at least partially account for the findings in this study, since we have not made determinations thereon. The endothelial dysfunction typically reported in this mouse model might represent a vulnerability trait addressing for cola drinking effects. A deregulation in the complex dialogue between mediators of inflammation, pro-coagulation, peroxidation, to mention a few, and the vascular system might be underlying present findings [20]–[22].

Diet habits, aging, gender and lipid profile negatively influence on the endothelial function in ApoE^−/−^mice [11] through the formation of reactive oxygen species. This scenario favors extensive lipid deposition in the major large arteries and the adhesion and transmigration of circulating monocytes into the aortic intima as well. The activated monocytes in the vascular wall are a source of pro-inflammatory and cytotoxic factors, inducing myofibroblasts' migration from the media layer and promoting extracellular matrix remodeling [23], [24].

ApoE^−/−^ mice that did not drink cola beverages (i.e.: W group) showed accelerated changes in atherosclerosis progression which paralleled a rapid increase in liver inflammation around the 20 weeks of age (Figure 3). These results concerning with the natural progression of atherosclerosis lesions in ApoE^−/−^ mice agree with those reported by Watson et al who found spontaneous acceleration of atherosclerotic lesions around the 20 weeks of life in this murine model as well [25]. The similar time dependence observed for the increase in aortic plaque area and liver inflammation in this study is consistent with a downstream expression of parallel alterations in gene expression in the aorta and liveras reported by other authors [26]. Taken together these findings emphasize the suggested key role for the liver in the process of atherogenesis at least in this murine model. Recently shared evidence confirms the existence of liver-artery interactions illustrating remote organ crosstalk in atherosclerosis [27].

### Conclusion

Consumption of cola beverages, regardless sugar content, increased the rate of atherosclerosis progression in ApoE^−/−^ mice favoring aortic plaque enlargement (inward remodeling) over media thinning. The effects of cola beverages drinking within the 8–16 weeks of age were not reversed long after cola discontinuation (30 weeks-old mice). Data suggest that cola beverages drinking during early life stages may accelerate worsening of atherosclerotic damagelater in life in a genetically favorable scenario, namely as found in the atherosclerosis-prone ApoE^−/−^ mice.

## Supporting Information

Figure S1 **Positive control of cytoplasmic immunostaining for CD3 in lymph node lymphocytes.** Magnification 400×.
(TIF)

Figure S2 **Positive control of nuclear immunostaining for PCNA in mitotically active neoplasic cells from an undifferentiated human sarcoma.** Magnification 400×.
(TIF)
