# Supplementary figures and images for: Rate of Atherosclerosis Progression in ApoE−/− Mice Long After Discontinuation of Cola Beverage Drinking
Source: PLoS One. 2014 Mar 26;9(3):e89838. doi: 10.1371/journal.pone.0089838 (PMC3966732; doi:10.1371/journal.pone.0089838)

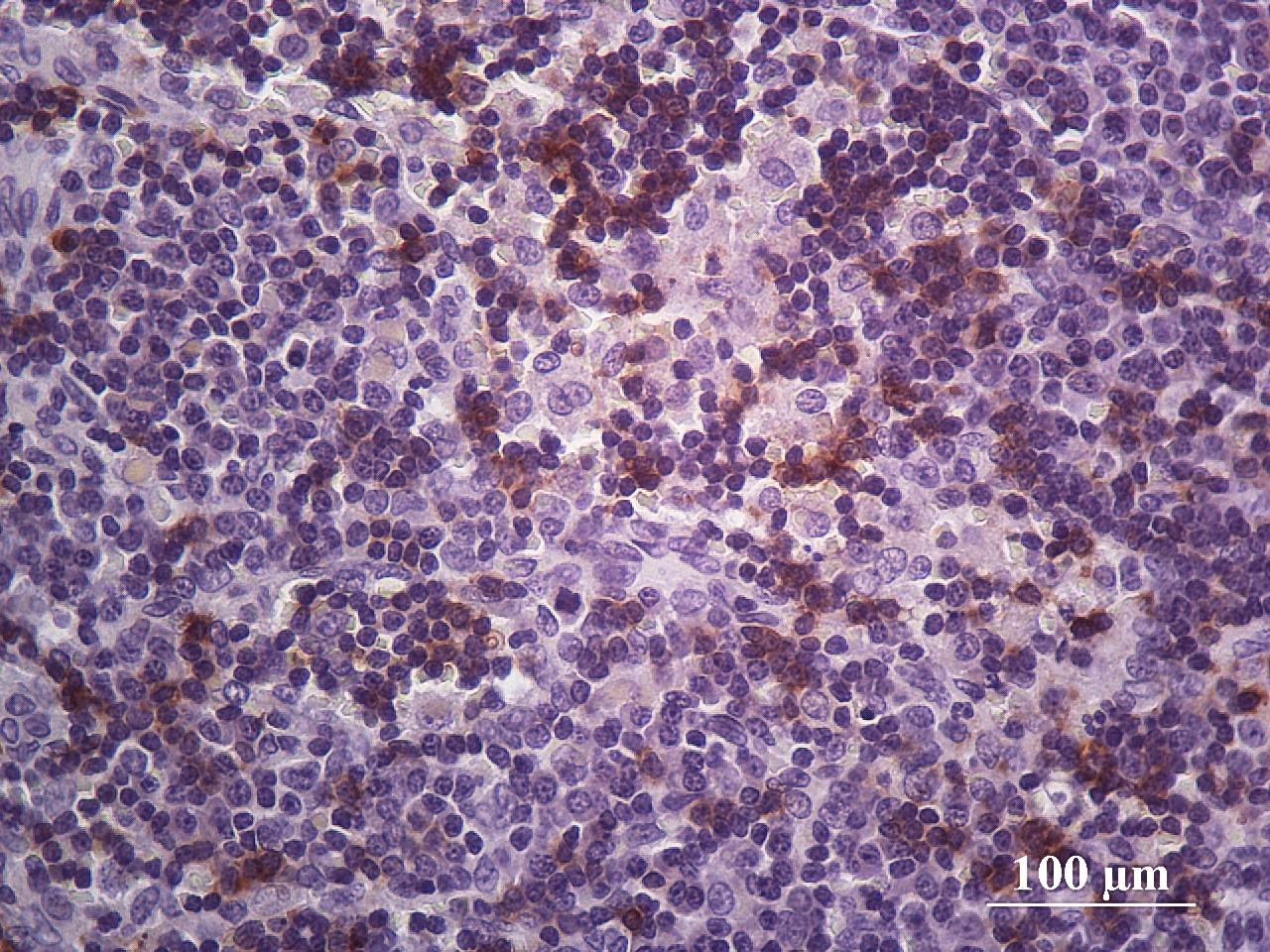

Supplement: Figure S1 — Positive control of cytoplasmic immunostaining for CD3 in lymph node lymphocytes. Magnification 400×. (TIF) [file pone.0089838.s001.tif]

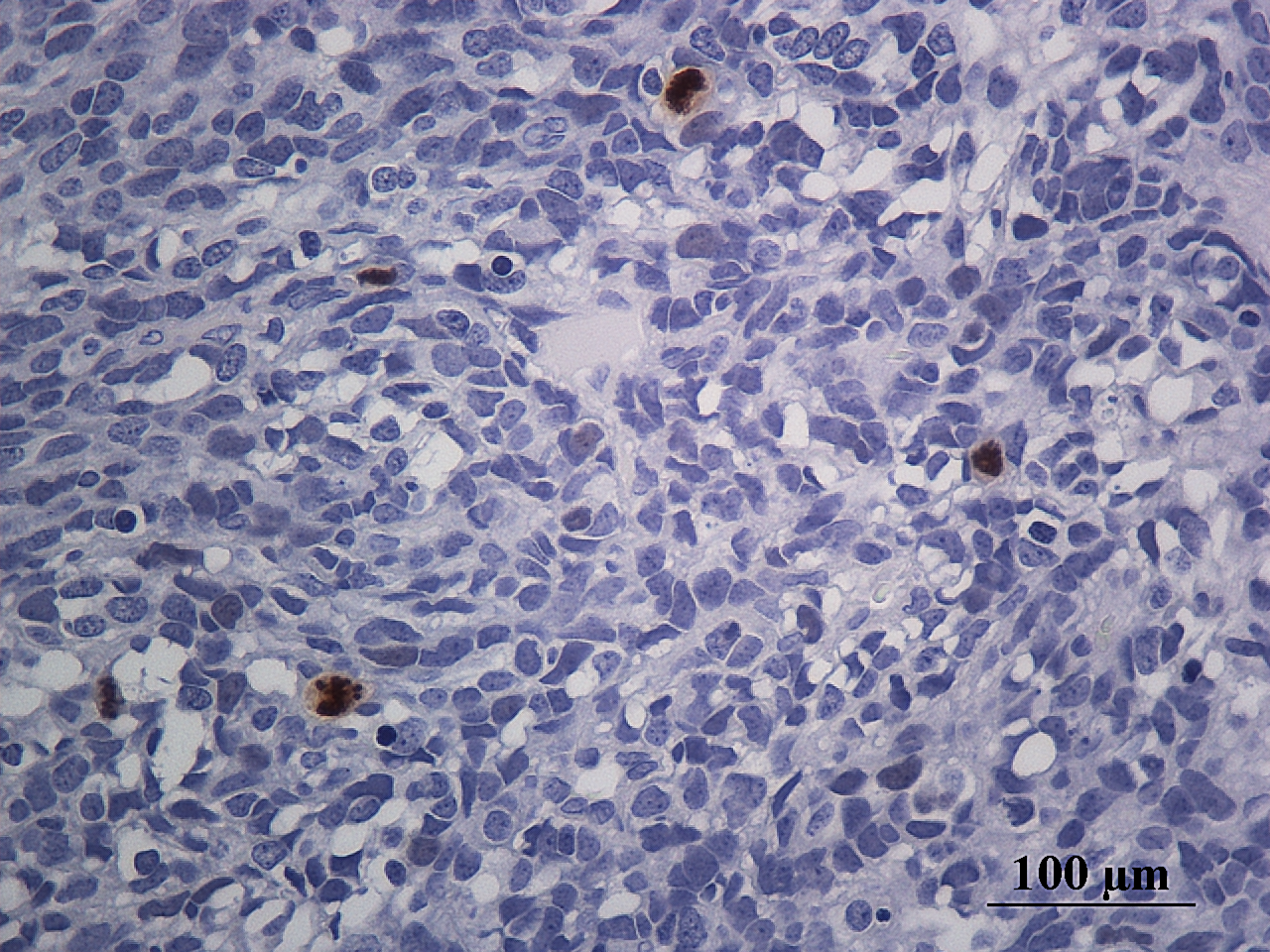

Supplement: Figure S2 — Positive control of nuclear immunostaining for PCNA in mitotically active neoplasic cells from an undifferentiated human sarcoma. Magnification 400×. (TIF) [file pone.0089838.s002.tif]
